# Microglia-associated research in Parkinson’s disease: a bibliometric analysis

**DOI:** 10.3389/fnagi.2025.1657349

**Published:** 2025-09-16

**Authors:** Yan-Jun Chen, Ming-Rong Xie, Sheng-Qiang Zhou, Fang Liu

**Affiliations:** ^1^Graduate School, Hunan University of Chinese Medicine, Changsha, China; ^2^National TCM Master Liu Zuyi Inheritance Studio, The Affiliated Hospital of Hunan Academy of Chinese Medicine, Changsha, China; ^3^The First Clinical College, Nanjing University of Chinese Medicine, Nanjing, China

**Keywords:** microglia, Parkinson’s disease, neuroinflammation, NLRP3 inflammasome, gut microbiota

## Abstract

**Background:**

Parkinson’s disease (PD) is a neurodegenerative disorder that predominantly affects the elderly. Evidence indicates that microglia-mediated neuroinflammation is recognized as one of the key mechanisms in PD. This study aims to analyze the key points, hotspots, and emerging frontiers in research related to PD and microglia.

**Method:**

Publications were obtained from the Web of Science and PubMed databases. VOSviewer and CiteSpace were used to generate visual representations and conduct numerical analyses of the dataset.

**Results:**

China and the United States were the leading contributors. Shanghai Jiao Tong University was the most active institution. The *Journal of Neuroinflammation* published the most papers on microglia and PD. Dr. Jau-Shyong Hong was the most prolific author. High-frequency keywords included PD, microglia, neuroinflammation, alpha-synuclein (a-syn), neurodegeneration, microglial activation, and oxidative stress. Gut microbiota and the NLRP3 inflammasome have garnered significant interest from researchers in recent years.

**Conclusion:**

This study generated visual mappings of microglia and PD-related research. Neuroinflammation, a-syn, neurodegeneration, microglial activation, and oxidative stress represent major focuses and hotspots in this field. Gut microbiota and the NLRP3 inflammasome have rapidly attracted research attention and are likely to be key directions for future studies in the coming years.

## 1 Introduction

Parkinson’s disease (PD) is a neurodegenerative disorder characterized by the progressive loss of dopaminergic neurons (Kalia and Lang, 2015). The pathogenesis of PD involves interactions among multiple factors, including genetic susceptibility, oxidative stress, and neuroinflammation (Morris et al., 2024; Ma et al., 2024). Although medications such as levodopa can alleviate symptoms by supplementing dopamine, current treatments are unable to halt disease progression, and long-term use may lead to motor complications (Aradi and Hauser, 2020). Additionally, significant challenges remain in managing non-motor symptoms and developing neuroprotective therapies, underscoring the urgent need to explore novel pathogenic mechanisms and therapeutic targets for PD.

Microglia maintain neural homeostasis under physiological conditions through dynamic monitoring of the microenvironment, clearance of metabolic waste, and synaptic pruning (Augusto-Oliveira et al., 2025). In response to acute injury, microglia adopt the M1 (pro-inflammatory) phenotype, releasing inflammatory factors to eliminate pathogens or necrotic tissue (Tang and Le, 2016). During the repair phase, they transition to the M2 (anti-inflammatory) phenotype, which promotes immunosuppression and tissue regeneration (Guo et al., 2022). However, in chronic neurodegenerative diseases such as PD, sustained microglial activation may result in excessive inflammatory responses and the release of neurotoxic substances, exacerbating neuronal damage (Lull and Block, 2010). This “double-edged sword” characteristic makes microglia dual modulators of both neuroprotection and neurodegenerative diseases.

Growing evidence indicates that microglial activation plays a pivotal role in PD. Activated microglia generate reactive nitrogen species and reactive oxygen species (ROS), contributing to mitochondrial dysfunction and neuronal oxidative damage (Block et al., 2007). They also facilitate the spread of alpha-synuclein (a-syn) via vesicle-mediated transport or cellular pathways (Guo et al., 2020). Single-cell transcriptomic analyses of midbrain tissues from PD patients have revealed a significant expansion of pro-inflammatory microglia, which correlates with dopaminergic neuron loss (Smajić et al., 2022). Furthermore, PD-associated gene mutations, such as those in *LRRK2* and *GBA1*, can alter microglial phagocytic function and inflammatory responses (Trainor et al., 2024). Investigating the relationship between microglia and PD is essential for advancing our understanding of the disease mechanisms and may provide breakthroughs for developing new treatments and personalized intervention strategies.

Bibliometrics involves the quantitative analysis of publication information, offering a data-driven basis for scientific policymaking and academic research (Ninkov et al., 2022). Although the number of publications on microglia and PD has grown with increasing research interest, there remains a lack of visual and analytical studies on research trends and focal points in this field. This study aims to identify key themes, hotspots, and emerging frontiers in PD and microglia-related research through a bibliometric approach.

## 2 Methods

### 2.1 Search strategy

**Figure 1:**
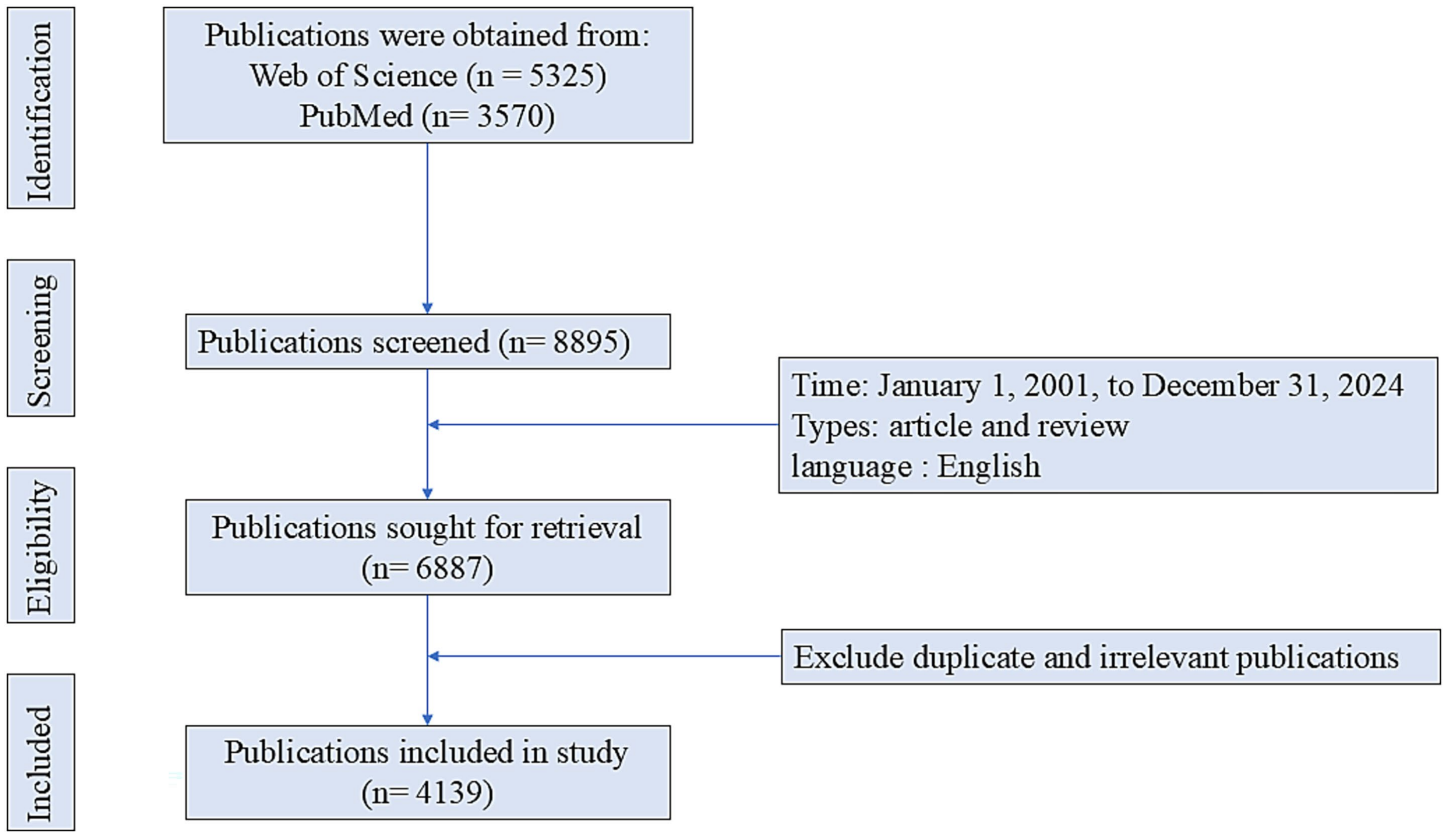
Literature search schematic diagram.

The primary data for this study were retrieved from the Web of Science (WoS) and PubMed databases. The specific search strategy in WoS was: (((TS = (microglia)) OR TS = (microglial cell)) OR TS = (microglial cells)) AND TS = (Parkinson’s disease). The search strategy applied in PubMed was: (((microglia [Title/Abstract]) OR (microglial cells [Title/Abstract])) OR (microglial cell [Title/Abstract])) AND (Parkinson’s disease [Title/Abstract]). Only articles and reviews published in English were included. The search period spanned from January 1, 1999, to December 31, 2024. Two independent investigators reviewed the records, removed duplicates, and excluded publications unrelated to the research topic. A total of 4,139 publications related to PD and microglia were ultimately included (Figure 1).

### 2.2 Software analysis

VOSviewer and CiteSpace were used to generate visual representations and perform quantitative evaluations of the dataset. The methodology is consistent with that described in previous literature (Chen et al., 2025a,b). VOSviewer, a software tool specializing in bibliometric clustering and visualization, facilitates the intuitive identification of academic research structures and hotspots by constructing collaboration networks and keyword co-occurrence maps (van Eck and Waltman, 2010). CiteSpace, widely employed in knowledge graph construction, enables the rapid detection of emerging trends and developmental pathways in research domains (Chen, 2004).

## 3 Results

### 3.1 Trend of publications

**Figure 2:**
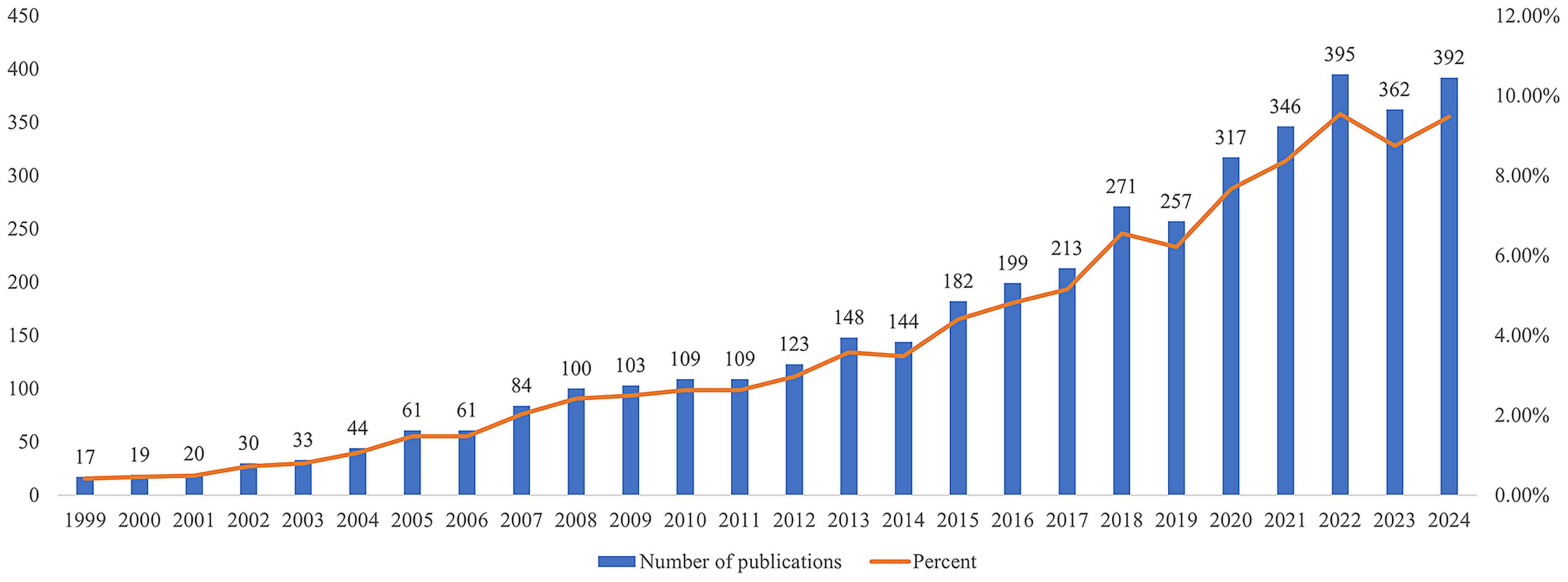
Publications on microglia and PD.

From 1999 to 2024, research on microglia and PD generally showed an upward trend (Figure 2). The slow growth phase (1999–2007) reflected the gradual recognition of the role of neuroinflammation in PD, representing a period of foundational knowledge accumulation. The accelerated growth phase (2008–2014) was likely attributed to the identification of pathological *α*-synuclein and the establishment of neuroinflammation as a central mechanism. The rapid growth phase (2015–2024) showed a marked increase, which was likely driven by multiple key factors, including targeted funding initiatives in neuroimmunology, in-depth investigations into neuroinflammatory mechanisms, and the application of novel technologies such as CRISPR gene editing and single-cell sequencing.

### 3.2 Country

**Figure 3:**
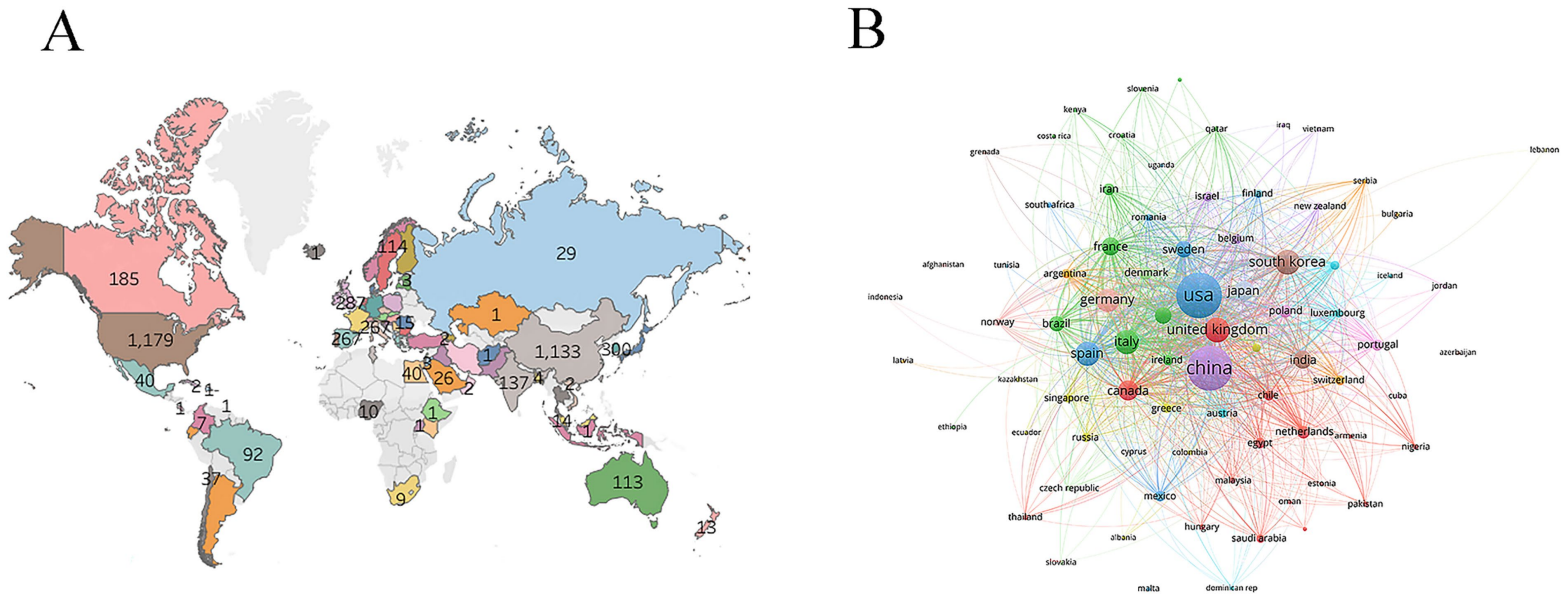
Countries. **(A)** Countries distribution. **(B)** Countries collaboration.

Studies related to microglia and PD were predominantly conducted in Asia and North America, with fewer contributions from South America (Figure 3A). Extensive collaborative networks were observed among different countries (Figure 3B). The United States and China led significantly in terms of publication output, ranking first and second with 1,179 and 1,133 papers, respectively (Table 1), highlighting their dominant roles in this field. The United States not only produced the highest number of publications but also achieved the highest total citations (89,641) and the highest average citations per paper (76.03), indicating a substantial academic impact. While China’s publication volume was comparable to that of the United States, its total citations (36,406) and average citations (32.13) were relatively lower, suggesting room for improvement in research influence. The leadership of the United States and China in microglia and PD research stems from a combination of factors. The leadership of the United States and China in microglia and PD research stems from a combination of factors. The United States benefits from long-term systematic investment in scientific research, world-leading research institutions and talent pools, a robust interdisciplinary collaboration framework, and strong capabilities in translating basic research—all contributing to high-quality output and academic impact. China’s progress is largely driven by national strategic support, including major scientific initiatives, consistently increasing research funding, and active recruitment of high-level talents, which together have rapidly expanded its research scale. Furthermore, increasingly intensive international collaborations have significantly enhanced the global visibility of Chinese research. Leveraging their respective strengths, the United States and China have established a dominant presence in this field worldwide.

**Table 1:** The top 10 countries.

Rank	Country	Publications	Citations	Average number of citations
1	USA	1,179	89,641	76.03
2	China	1,133	36,406	32.13
3	South Korea	300	15,359	51.20
4	United Kingdom	287	19,548	68.11
5	Italy	267	13,433	50.31
6	Spain	267	13,713	51.36
7	Germany	251	14,871	59.25
8	Canada	185	10,939	59.13
9	Japan	164	7,772	47.39
10	France	138	9,715	70.40

### 3.3 Institutions

**Figure 4:**
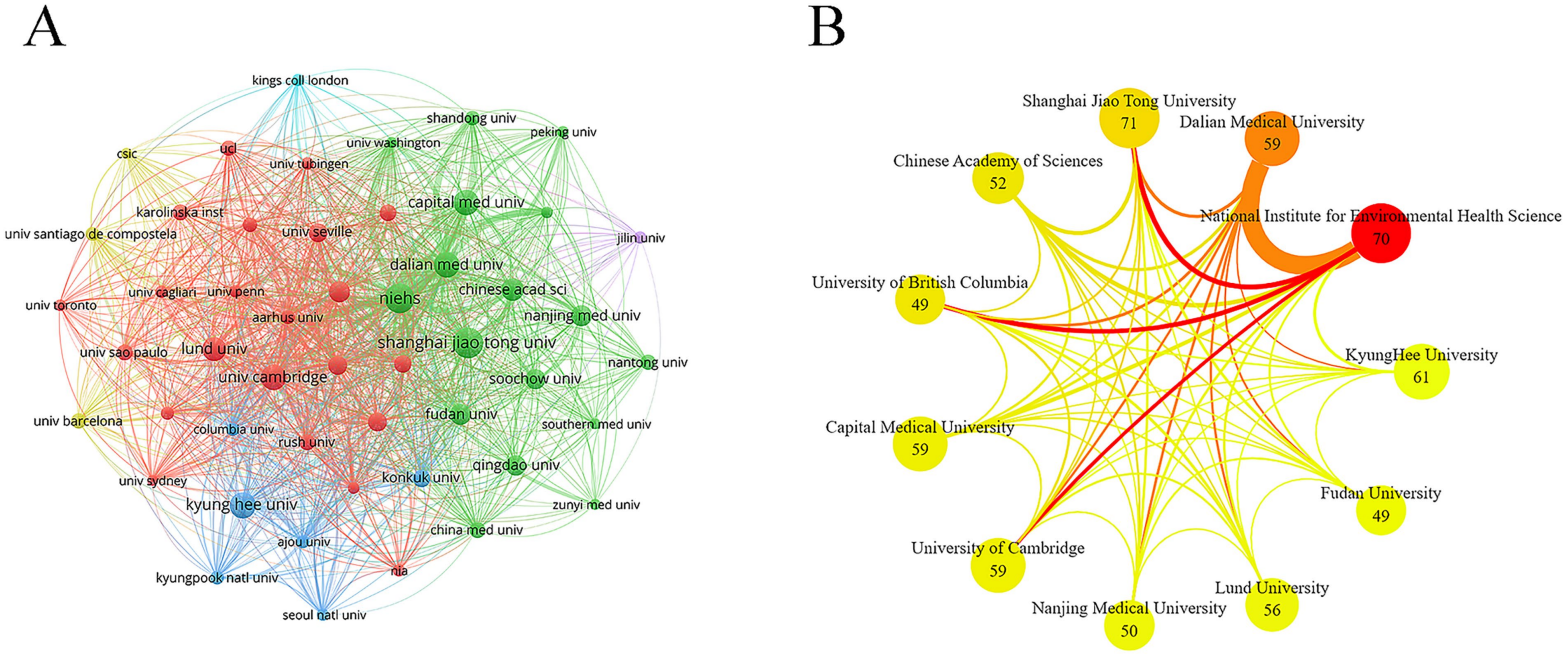
Institutions. **(A)** Institutions collaboration. **(B)** Top 10 institutions network collaboration.

Collaborative relationships were observed among research institutions across different countries (Figure 4A). Shanghai Jiao Tong University ranked first in terms of publication output, with 71 papers, demonstrating strong research productivity in this area. The National Institute of Environmental Health Sciences ranked second in publication count. It also achieved notably higher total citations (11,347) and average citations (162.10) than other institutions, reflecting the high impact and broad recognition of its research. Additionally, Dalian Medical University and the University of British Columbia, despite having relatively fewer publications (59 and 49, respectively), excelled in total and average citations, indicating high research quality. Chinese institutions accounted for six of the listed entities, reflecting a quantitative advantage, though their overall influence still lagged behind that of several European and American institutions (Table 2). In the institutional collaboration network, line thickness represents the strength of collaborative ties. Among the top ten most active institutions (Figure 4B), Dalian Medical University exhibited the closest collaboration with the National Institute of Environmental Health Sciences. Leading academic institutions in China and the United States occupy dominant positions in microglia and PD research. This prominence results from highly efficient scientific research ecosystems in both countries, albeit through different mechanisms. China’s success is underpinned by concentrated resource allocation aligned with national strategies, abundant clinical resources, and effective large-scale collaborative efforts. The United States’ advantages include a culture of open scientific inquiry, sustained support for high-risk basic research, and a tradition of pioneering paradigm-shifting innovations. These complementary factors collectively facilitate the production of high-quality and high-impact research.

**Table 2:** The top 10 institutions.

Rank	Institution	Documents	Citations	Average number of citations
1	Shanghai Jiao Tong University	71	2,947	41.51
2	National Institute for Environmental Health Science	70	11,347	162.10
3	KyungHee University	61	2,968	48.66
4	Capital Medical University	59	1,406	23.83
5	Dalian Medical University	59	5,878	99.63
6	University of Cambridge	59	2,597	44.02
7	Lund University	56	3,328	59.43
8	Chinese Academy of Sciences	52	4,130	79.42
9	Nanjing Medical University	50	2,491	49.82
10	Fudan University	49	1,426	29.10
10	University of British Columbia	49	5,001	102.06

### 3.4 Journals and co-cited journals

**Figure 5:**
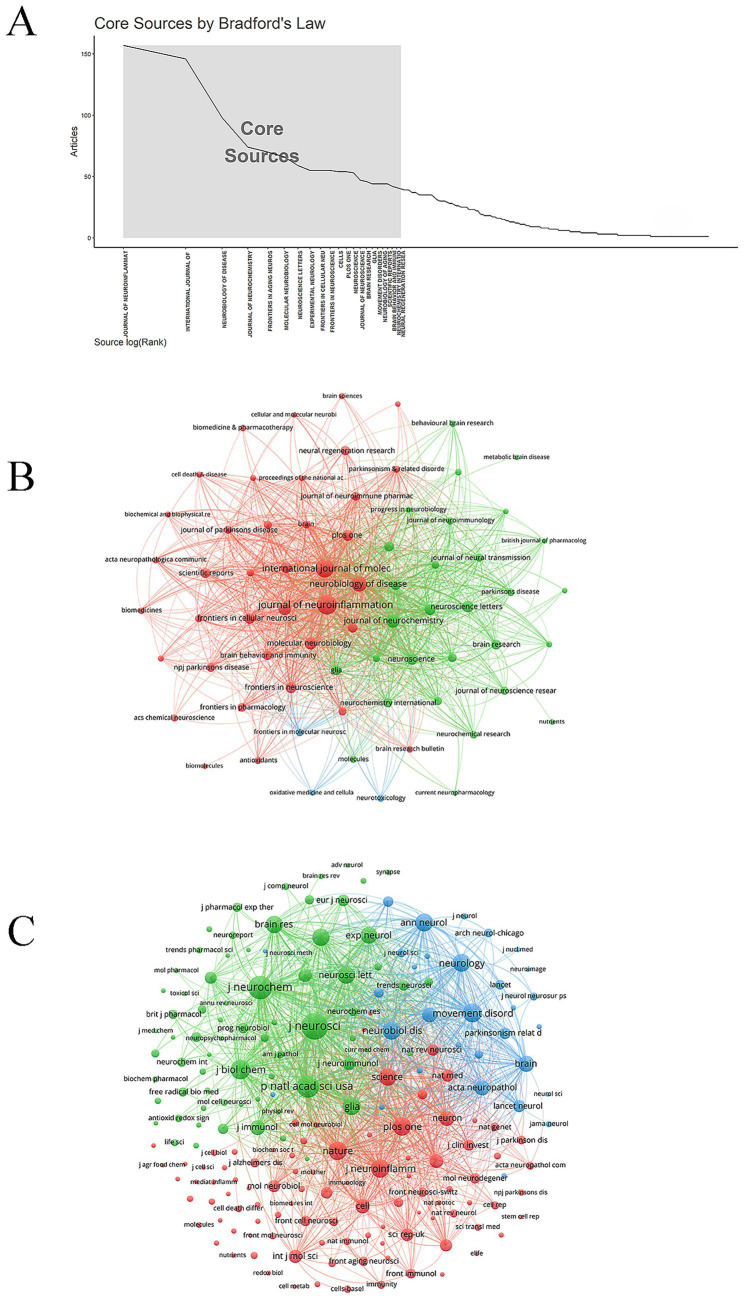
Journals and co-cited journals. **(A)** Core journal. **(B)** Journal network. **(C)** Co-cited journal network.

A total of 22 core journals were identified based on Bradford’s law (Figure 5A). The common focus of these core journals emphasized multi-level research approaches—from molecular and cellular to systemic levels—integrating multidisciplinary methodologies such as immunology, metabolism, and bioinformatics to elucidate the complex mechanisms underlying neurological diseases. The *Journal of Neuroinflammation* published the most articles on microglia and PD (157 papers). Notably, the *Journal of Neurochemistry* had the highest average citation count (98.08 citations), indicating considerable academic influence (Figure 5B; Table 3). The top three co-cited journals were the *Journal of Neuroscience*, the *Journal of Neurochemistry*, and the *Proceedings of the National Academy of Sciences of the United States of America*, with 10,516, 8,249, and 7,770 citations, respectively (Figure 5C). The top ten co-cited journals were all high-quality publications, with eight ranked in Q1 and two in Q2 (Table 4).

**Table 3:** The top 10 journals.

Rank	Source	Documents	Citations	Average number of citations	IF	JCR
1	Journal of Neuroinflammation	157	7,670	48.85	9.3	Q1
2	International Journal of Molecular Sciences	146	3,512	24.05	4.9	Q1
3	Neurobiology of Disease	98	7,399	75.50	5.1	Q1
4	Journal of Neurochemistry	74	7,258	98.08	4.2	Q2
5	Frontiers in Aging Neuroscience	70	2,455	35.07	4.1	Q2
6	Molecular Neurobiology	66	4,035	61.14	4.6	Q1
7	Neuroscience Letters	59	2,137	36.22	2.5	Q3
8	Experimental Neurology	55	3,640	66.18	4.6	Q1
8	Frontiers In Cellular Neuroscience	55	2,965	53.91	4.2	Q2
8	Frontiers in Neuroscience	55	1926	35.02	3.2	Q2

**Table 4:** The top 10 co-cited journals.

Rank	Source	Citations	IF	JCR
1	Journal of Neuroscience	10,516	4.4	Q1
2	Journal of Neurochemistry	8,249	4.2	Q2
3	Proceedings of The National Academy of Sciences of The United States of America	7,770	9.4	Q1
4	Journal of Biological Chemistry	5,855	4	Q2
5	Movement Disorders	5,434	7.4	Q1
6	Nature	5,323	50.5	Q1
7	Neurobiology of Disease	5,173	5.1	Q1
8	Journal of Neuroinflammation	5,065	9.3	Q1
9	Plos One	5,063	2.9	Q1
10	Neurology	4,986	8.4	Q1

### 3.5 Authors and co-cited authors

**Figure 6:**
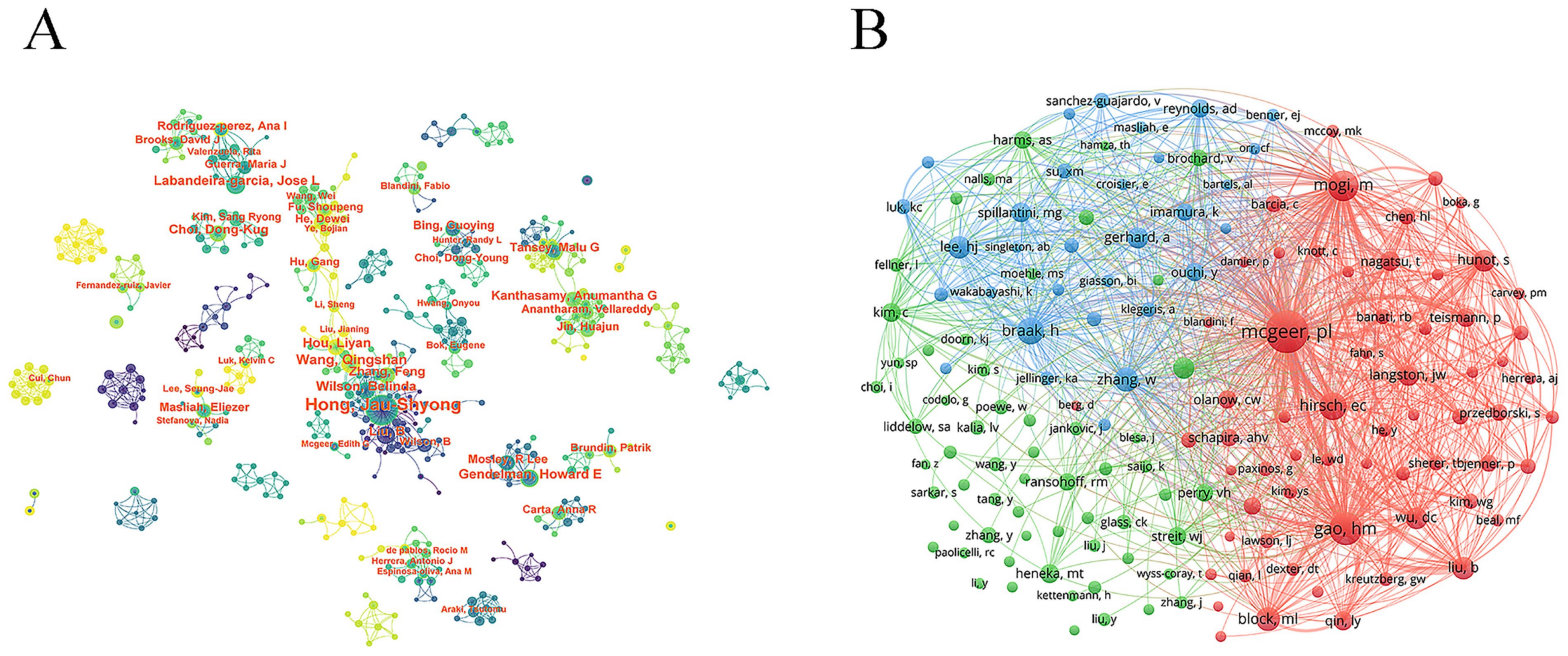
Authors and co-cited authors. **(A)** Authors collaboration. **(B)** Co-cited authors collaboration.

Highly productive authors are often leading figures in the research field. Dr. Jau-Shyong Hong from the National Institute of Environmental Health Sciences was the most prolific author, with 75 publications, followed by Dr. Qingshan Wang from Dalian Medical University (30 publications) and Dr. Jose L. Labandeira-Garcia from Universidad de Santiago de Compostela (28 publications) (Table 5). Notably, Dr. Malu G. Tansey from the University of Florida had the highest average number of citations (154.92), reflecting the broad impact of her academic work. These productive authors maintained stable partnerships and established research teams (Figure 6A). Co-cited author analysis reveals academic connections among researchers. Dr. Patrick L. McGeer, Dr. Hui-Ming Gao, and Dr. Masaki Mogi were the top three co-cited authors, with 2,127, 1,224, and 1,088 citations, respectively (Figure 6B; Table 6).

**Table 5:** The top 10 authors.

Rank	Author	Documents	Citations	Average number of citations	Country	Institution
1	Dr. Hong, Jau-Shyong	75	10,902	145.36	USA	NIH National Institute of Environmental Health Sciences
2	Dr. Wang, Qingshan	30	991	33.03	China	Dalian Medical University
3	Dr. Labandeira-Garcia, Jose L.	28	1,400	50.00	Spain	Universidad de Santiago de Compostela
4	Dr. Gendelman, Howard E.	27	3,071	113.74	USA	University of Nebraska Medical Center
4	Dr. Tansey, Malu Gamez	26	4,028	154.92	USA	University of Florida
6	Dr. Wilson, Belinda	25	2,119	84.76	USA	NIH National Institute of Environmental Health Sciences
7	Dr. Choi, Dong-Kug	23	1,083	47.09	South Korea	Konkuk University
8	Dr. Kanthasamy, Anumantha G.	23	1704	74.09	USA	Iowa State University
9	Dr. Hou, Li-Yan	22	654	29.73	China	Chinese Academy of Medical Sciences
10	Dr. Mosley, R. Lee	22	2,712	123.27	USA	University of Nebraska Medical Center
10	Dr. Rodriguez Perez Ana Isabel	22	1,021	46.41	Spain	Complexo Hospitalario Universitario de Santiago de Compostela

**Table 6:** The top 10 co-cited authors.

Rank	Author	Citations	Country	Institution
1	Dr. Mcgeer, Patrick L.	2,127	Canada	University of British Columbia
2	Dr. Gao, Hui-Ming	1,224	USA	NIH National Institute of Environmental Health Sciences
3	Dr. Masaki Mogi	1,088	Japan	Ehime University
4	Dr. Hirsch, Etienne C.	880	France	Sorbonne University
5	Dr. Braak, Heiko	849	Germany	Ulm University
6	Dr. Zhang, Wei	702	USA	NIH National Institute of Environmental Health Sciences
6	Dr. Block, Michelle L.	696	USA	Indiana University System
8	Dr. Liu, Bin	626	China	North China University of Science & Technology
9	Dr. Lee, Hyuk-Joon	596	USA	Duke University
10	Dr. Wu, Dougias C.	565	China	Beijing Forestry University

### 3.6 Co-cited references

**Figure 7:**
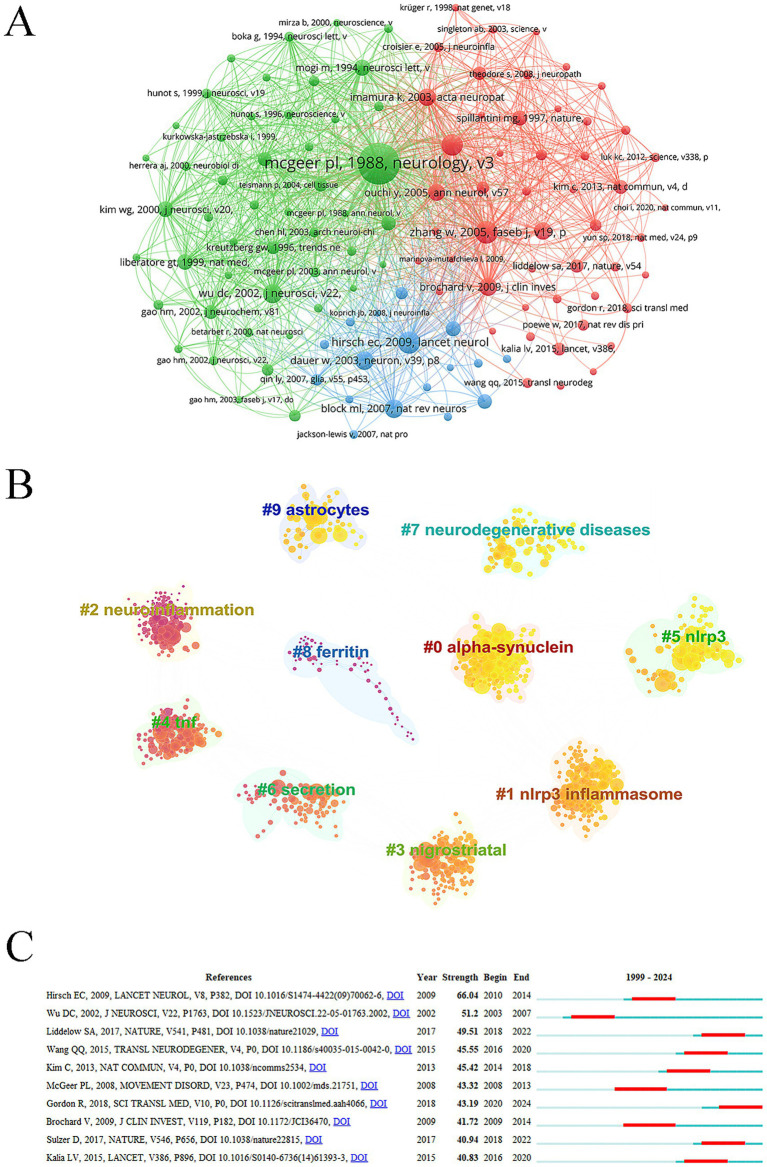
Co-cited references. **(A)** References network. **(B)** Co-cited reference cluster analysis. **(C)** Co-cited references burst.

Highly co-cited references typically represent foundational studies in the field. The article entitled “*Reactive microglia are positive for HLA-DR in the substantia nigra of Parkinson’s and Alzheimer’s disease brains* (McGeer et al., 1988),” published in *Neurology*, received the highest number of co-citations (Figure 7A). This study identified abundant HLA-DR-positive reactive microglia in the substantia nigra of patients with PD and Alzheimer’s disease, along with the presence of Lewy bodies and free melanin. Although less pronounced, similar pathological changes were observed in the substantia nigra of most Alzheimer’s disease cases, and extensive hippocampal involvement was noted in dementia cases. These findings indicate that HLA-DR-positive reactive microglia are sensitive indicators of neuropathological activity and suggest co-occurrence of PD and Alzheimer’s disease pathology in elderly patients. The most frequently cited references primarily focused on PD, particularly mechanisms involving microglia in disease pathogenesis (Table 7). Reference clustering, visualized by color gradient from dark to light, reflected the evolution of the research foundation, shifting from early themes such as neuroinflammation and TNF to more recent foci including NLRP3 and a-syn (Figure 7B). The paper titled “*Inflammasome inhibition prevents α-synuclein pathology and dopaminergic neurodegeneration in mice* (Gordon et al., 2018)” exhibited the highest citation burst intensity between 2020 and 2024, indicating a rapidly growing influence (Figure 7C). This study, published in 2018, systematically demonstrated the central role of the NLRP3 inflammasome in PD pathology. NLRP3 activation was observed in the substantia nigra of PD patients and in various PD models—including those induced by *α*-syn fibrils and mitochondrial dysfunction—as an early event triggering the release of IL-1β and ASC. Mechanistic investigations revealed that α-synuclein fibrils specifically activate the NLRP3 pathway, provoking a robust inflammatory response. Importantly, the authors showed that the oral NLRP3 inhibitor MCC950 effectively crosses the blood–brain barrier, suppresses inflammasome activation, and ameliorates motor deficits, dopaminergic neurodegeneration, and α-syn pathology in animal models, thereby validating NLRP3 as a promising therapeutic target for PD.

**Table 7:** The top 10 references.

Rank	Title	Type	Citation times	Year	Journal	IF	JCR
1	Reactive microglia are positive for HLA-DR in the substantia nigra of Parkinson’s and Alzheimer’s disease brains.	Article	962	1988	Neurology	8.4	Q1
2	Aggregated α-synuclein activates microglia: a process leading to disease progression in Parkinson’s disease	Article	443	2005	Faseb Journal	4.4	Q2
3	Neuroinflammation in Parkinson’s disease: a target for neuroprotection?	Review	436	2009	Lancet Neurology	46.6	Q1
4	In vivo imaging of microglial activation with [11C](R)-PK11195 PET in idiopathic Parkinson’s disease	Article	423	2006	Neurobiology of Disease	5.1	Q1
5	Blockade of microglial activation is neuroprotective in the 1-methyl-4-phenyl-1,2,3,6-tetrahydropyridine mouse model of Parkinson disease	Article	360	2002	Journal of Neuroscience	4.4	Q1
5	Microglia-mediated neurotoxicity: uncovering the molecular mechanisms	Review	353	2007	Nature Reviews Neuroscience	28.7	Q1
7	Parkinson’s disease: Mechanisms and models	Review	351	2003	Neuron	14.7	Q1
8	Infiltration of CD4 + lymphocytes into the brain contributes to neurodegeneration in a mouse model of Parkinson disease	Article	340	2009	Journal of Clinical Investigation	13.3	Q1
9	Staging of brain pathology related to sporadic Parkinson’s disease	Review	322	2003	Neurobiology of Aging	3.7	Q2
10	Distribution of major histocompatibility complex class II-positive microglia and cytokine profile of Parkinson’s disease brains	Article	318	2003	Acta Neuropathologica	9.3	Q1

### 3.7 Keywords analysis

**Figure 8:**
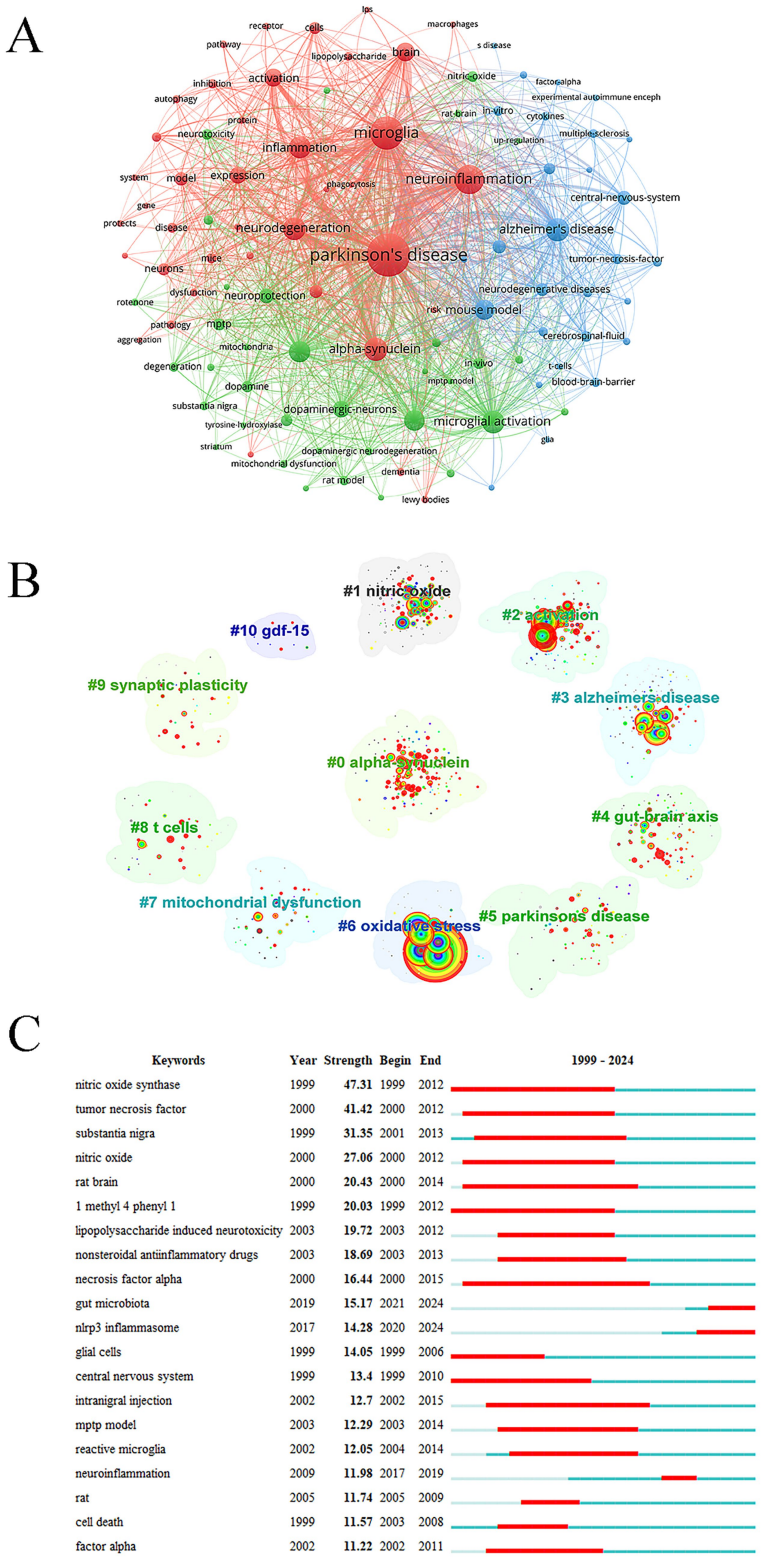
Keywords. **(A)** Keywords network. **(B)** Keywords cluster analysis. **(C)** Keywords burst.

High-frequency keywords serve as important indicators of research hotspots, thematic evolution, and knowledge structure within a scientific domain. The most frequent keywords in microglia and PD research included: PD (2,863 times), microglia (1,765 times), neuroinflammation (1,352 times), a-syn (902 times), neurodegeneration (843 times), microglial activation (838 times), and oxidative stress (737 times) (Figure 8A). Each keyword cluster represented a relatively independent research topic or subfield, reflecting core research directions. Cluster #2 activation highlighted the focus on neuro-immune responses mediated by microglia in PD. Clusters #6 oxidative stress and #7 mitochondrial dysfunction indicated that microglial activation was closely associated with key cellular damage mechanisms, collectively driving neurodegenerative processes in PD. Cluster #3 Alzheimer’s disease aligned with conclusions from highly co-cited references, confirming significant overlap and co-occurrence of neuroinflammatory mechanisms between PD and Alzheimer’s disease, representing an important interdisciplinary research area. Cluster #4 gut-brain axis reflected a contemporary research hotspot, suggesting that the gut microbiome may influence microglial states via immune and inflammatory pathways, thereby contributing to PD pathogenesis. Cluster #8 T cells indicated that research has expanded beyond innate immunity (microglia) to include adaptive immune mechanisms (T cells) and their interactions with microglia in shaping the neuroinflammatory environment (Figure 8B). Keywords with the strongest citation bursts between 2020 and 2024 were gut microbiota and NLRP3 inflammasome, reflecting rapidly growing research interest in these topics (Figure 8C).

## 4 Discussion

### 4.1 General information

From 1999 to 2024, research on microglia and PD exhibited a general upward trend. China and the United States emerged as the leading contributors in this field. Shanghai Jiao Tong University was the most productive institution. The *Journal of Neuroinflammation* published the highest number of papers on microglia and PD. Dr. Jau-Shyong Hong was the most prolific author. Extensive collaboration among a wide range of institutions and researchers facilitated advancements in this area.

### 4.2 Hotspots and frontiers

**Figure 9:**
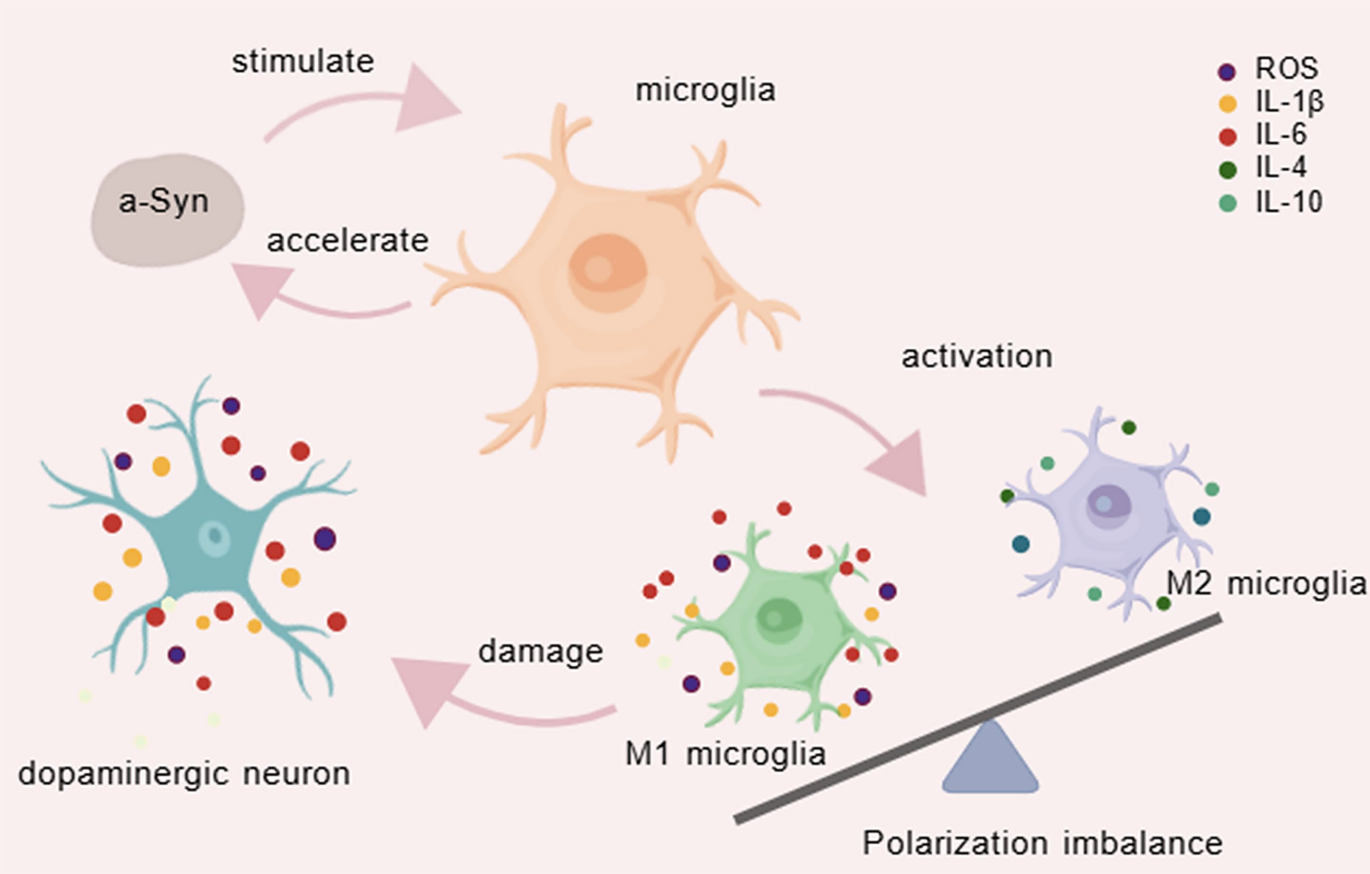
The mechanism diagram of microglia in PD.

In addition to PD and microglia, other extensively studied topics included neuroinflammation, a-syn, neurodegeneration, microglial activation, and oxidative stress (Figure 9). Recently, gut microbiota and NLRP3 inflammasome have exhibited the strongest citation bursts, reflecting growing interest and suggesting that these topics represent key frontiers for future research.

In the activated state, microglia exhibit enlarged cell bodies, shortened processes, and migration toward sites of injury or pathology (Kreutzberg, 1996). Microglia-mediated neuroinflammation is a central mechanism in PD pathogenesis (Isik et al., 2023). Activated microglia perpetuate neuroinflammation through continuous release of IL-1β and IL-6, which exacerbate inflammatory responses, induce neuronal apoptosis, and inhibit neural regeneration (Badanjak et al., 2021). The gene encoding a-syn, *SNCA*, is associated with familial forms of PD (Yaribash et al., 2025). The abnormal aggregation of a-syn represents a hallmark of PD pathology (Burré et al., 2024). A self-reinforcing vicious cycle exists between a-syn and microglia: aberrant a-syn aggregates are recognized as danger signals by microglial TLR2, triggering inflammatory activation (Kim et al., 2013). Inflammatory mediators can upregulate a-syn expression, promote its abnormal phosphorylation, and facilitate its aggregation and spread (Harackiewicz and Grembecka, 2024). Microglia serve as both a primary source of and a responder to oxidative stress. Through the membrane-bound enzyme NADPH oxidase, microglia generate excessive ROS, contributing to dopaminergic neuron damage (Qin et al., 2004). Furthermore, mitochondrial electron transport chain defects in activated microglia lead to ROS leakage, which can propagate pro-inflammatory signals and induce mitochondrial dysfunction in neurons (Dias et al., 2013). Significant differences in gut microbiota composition have been observed between PD patients and healthy individuals (Unger et al., 2016). The gut-brain axis provides a critical communication pathway through which gut microbiota and microglia interact in PD. Microbial metabolites, such as short-chain fatty acids, can modulate microglial function, attenuate neuroinflammatory responses, and promote microglial homeostasis (Erny et al., 2015). a-syn activates the NLRP3 inflammasome by disrupting lysosomal membranes, inducing mitochondrial dysfunction and plasma membrane depolarization, thereby enhancing IL-1β production and exacerbating neuroinflammation (Huang et al., 2023). Inhibition of the NLRP3 inflammasome has been shown to block a-syn-induced neuronal death (Gordon et al., 2018). In microglia, mitochondrial impairment amplifies NLRP3 inflammasome signaling, further aggravating dopaminergic neurodegeneration (Sarkar et al., 2017).

### 4.3 Research challenges and future directions

The role of microglia in PD involves a specific mechanism, centered on the bidirectional interaction between *α*-synuclein and microglia. Extracellular aggregated α-syn serves as a major damage-associated molecular pattern that activates microglia in PD. It promotes neuroinflammation by engaging pro-inflammatory pathways such as NF-κB through pattern recognition receptors, including TLR2 and TLR4 (Fellner et al., 2013). The mutant form of LRRK2 represents one of the most common genetic risk factors for PD. It is expressed not only in neurons but is also highly enriched in microglia. *LRRK2* enhances microglial inflammatory responses and impairs phagocytic function (Kim et al., 2018). The *GBA1* mutation is the strongest genetic risk factor for PD. *GBA1* can lead to the loss of function of the encoded glucocerebrosidase, disrupt the homeostasis of microglia cells, promote the generation of pro-inflammatory phenotypes, and create a microenvironment conducive to the aggregation and spread of *α*-syn (Do et al., 2019). Although targeting microglia has emerged as a highly promising strategy for disease-modifying therapies in PD, the development of anti-inflammatory drugs directed at microglia remains limited, with relatively few related clinical trials. MCC950, a potent and selective NLRP3 inhibitor, has been shown in PD mouse models to effectively suppress NLRP3 activation and IL-1β production. It significantly reduces microglial activation, prevents dopaminergic neuron loss and *α*-syn pathology spread, and improves motor function (Gordon et al., 2018). Pioglitazone, a PPARγ agonist used in type 2 diabetes, exhibits anti-inflammatory properties. In MPTP-induced PD mouse models, it inhibits microglial activation, reduces pro-inflammatory cytokine release, and demonstrates notable neuroprotective effects (Breidert et al., 2002). These promising preclinical outcomes led to its advancement into clinical trials. However, a phase II randomized, double-blind, placebo-controlled trial of pioglitazone failed to meet its primary endpoint (NINDS Exploratory Trials in Parkinson Disease (NET-PD) FS-ZONE Investigators, 2015). Compared to placebo, the 210 early-stage PD patients receiving pioglitazone did not show statistically significant delays in disease progression. This underscores the challenges in translating findings from animal studies to human applications, which may be influenced by factors such as patient selection, treatment timing, and dosage.

Several challenges remain in current research. Microglia can clear *α*-synuclein and cellular debris but may also induce oxidative stress and neuronal damage upon overactivation. The functional complexity of microglia stems from their heterogeneity and plasticity. At different stages of PD, microglia may adopt either pro-inflammatory or neuroprotective phenotypes, and their roles may vary considerably across subtypes. Thus, personalized research strategies are warranted. PD patients exhibit high heterogeneity in genetic background and pathological stage, complicating the translation from animal models to humans. Inflammatory responses in neurotoxin-based animal models may not fully recapitulate the slow, *α*-synuclein-driven neuroinflammation characteristic of human PD.

Future research should prioritize the following directions. Application of spatial transcriptomics and single-cell multi-omics to characterize disease-specific microglial substates in human PD brain samples. Development of advanced human iPSC-derived microglial models to simulate neuro-immune interactions in PD *in vitro* and to enable high-throughput drug screening. Prospective stratification of research cohorts based on clinical subtypes, genetic background, and neuroinflammatory biomarkers. Functional comparison of iPSC-derived microglia from different patient subgroups, or use of microglia-targeted PET imaging to analyze *in vivo* activation status, drivers, and contributions of specific microglial subpopulations to disease progression. The NLRP3 inflammasome serves as a central node linking mitochondrial dysfunction, *α*-syn pathology, and neuroinflammation, representing a promising therapeutic target. Administration of anti-NLRP3 agents in pre-disease models to assess their potential in preventing neuronal loss and pathological spread. Exploration of surrogate markers for monitoring NLRP3 activation to facilitate patient screening and pharmacodynamic evaluation in clinical trials. Evaluation of synergistic effects between NLRP3 inhibitors and α-syn-targeted therapies or mitochondrial protective agents to enable multi-pathway intervention. Gut microbiota dysbiosis is a potential initiator of PD, and its role in central nervous inflammation via the gut-brain axis is a topic of active investigation. Examining the direct effects of specific microbial metabolites (e.g., short-chain fatty acids) on microglial maturation and function. Supplementing probiotics or prebiotics in PD animal models and evaluating their effects on microglial phenotype, α-syn clearance, and motor symptom improvement. Through more precise and in-depth analyses of microglia, we may not only uncover novel mechanisms of PD but also pave the way for developing disease-modifying therapies that can prevent or slow disease progression.

### 4.4 Limitations

This study has several limitations. First, the literature analysis was restricted to articles published in English. Studies in other languages or of other types may not have been captured. Second, database selection was limited to Web of Science (WOS) and PubMed; other databases such as Scopus and Embase were not included, which may have resulted in the omission of relevant literature. Third, the search period spanned from January 1, 1999, to December 31, 2024. Some recently published studies may not have been identified due to insufficient time for accumulation and indexing.

## 5 Conclusion

This study generated visual mappings of microglia and PD-related research. Neuroinflammation, a-syn, neurodegeneration, microglial activation, and oxidative stress represent major focuses and hotspots in this field. Gut microbiota and the NLRP3 inflammasome have rapidly attracted research attention and are likely to be key directions for future studies in the coming years.

## Data Availability

The original contributions presented in the study are included in the article/supplementary material, further inquiries can be directed to the corresponding authors.
